# A New Era of Laparoscopic Revision of Kasai Portoenterostomy for the Treatment of Biliary Atresia

**DOI:** 10.1155/2015/173014

**Published:** 2015-07-22

**Authors:** Naruhiko Murase, Hiroo Uchida, Yasuyuki Ono, Takahisa Tainaka, Kazuki Yokota, Akihide Tanano, Chiyoe Shirota, Ryo Shirotsuki

**Affiliations:** Department of Pediatric Surgery, Nagoya University Graduate School of Medicine, 65 Tsurumai, Showa, Nagoya 466-8550, Japan

## Abstract

*Purpose.* Kasai portoenterostomy is the standard therapy for biliary atresia (BA). If Kasai is unsuccessful, there is controversy over whether revision of Kasai restores adequate biliary drainage. Although there are several reports of laparoscopic Kasai (Lap-Kasai), none has described laparoscopic revision (Lap-revision). The purposes of this study were to evaluate the feasibility and efficacy of Lap-revision. *Methods.* 65 patients underwent open Kasai between November 2001 and November 2013, and 12 patients underwent Lap-Kasai between December 2013 to January 2015. The indications for revision included bile flow cessation and recurrent cholangitis. Clinical data were compared between open and laparoscopic revisions of Kasai. *Results.* Open revision of Kasai was performed in 20 patients after open Kasai, and Lap-revision was performed in 4 patients after Lap-Kasai. Lap-revision was completed without conversion or major complication in any patient. The bilirubin level was normalized by Lap-revision in all four patients, and three of them were alive with their native liver. Open and laparoscopic revisions of Kasai were comparable in terms of the operation time, blood loss, and surgical outcomes. *Conclusion.* Lap-revision is a feasible and effective method for the treatment of BA and might herald a new era for the treatment of this disease.

## 1. Introduction

Biliary atresia (BA) is a progressive fibroobliterative disorder that results in cirrhosis or end-stage liver disease [1]. Although liver transplantation (LT) has greatly improved the prognosis of patients with BA, Kasai portoenterostomy (Kasai) is still regarded as the standard initial therapy because of the medical costs associated with LT and the shortage of eligible donors [1, 2]. Kasai is unsuccessful in some patients, but the necessity and indications for revision of Kasai are controversial [2–6]. Although revision of Kasai could achieve adequate biliary drainage, surgeons may avoid repeated abdominal procedures to treat BA because they may have a negative effect on the outcomes of subsequent LT [5, 6]. Revision of Kasai via laparotomy is frequently performed in our institution, and the native liver is preserved by revision of Kasai in some patients. Therefore, we are certain that revision of Kasai is a valuable treatment option in the era of LT.

Although there are several reports of laparoscopic Kasai (Lap-Kasai), there are no reports describing the outcomes of laparoscopic revision of Kasai (Lap-revision). We first performed Lap-Kasai in December 2013, and Lap-revision is now performed as frequently as open revision of Kasai. In this report, we present four patients who underwent Lap-revision. We performed a retrospective analysis to compare the clinical data between open and laparoscopic revision of Kasai to examine the feasibility and efficacy of Lap-revision in patients with BA.

## 2. Methods

### 2.1. Patients

We retrospectively reviewed the medical records of patients who were diagnosed with BA and who underwent Kasai at our institution between November 2001 and January 2015. Between November 2001 and November 2013, 65 patients underwent open Kasai. Lap-Kasai was introduced in December 2013, and 12 patients underwent Lap-Kasai between December 2013 and January 2015. There were no exclusion criteria for laparoscopic procedure.

The indications for revision of Kasai include bile flow cessation and recurrent cholangitis. We performed revision of Kasai after providing the patients' parents with a sufficient explanation of the treatment options, including LT, and obtaining their consent. In this study, revision of Kasai was conducted via laparotomy if Kasai was done via laparotomy. Conversely, patients whose Kasai was done laparoscopically underwent Lap-revision.

### 2.2. Surgical Techniques

The basic procedures were essentially the same regardless of whether the procedure was done via laparotomy or laparoscopy. Our laparoscopic techniques are described in more detail below.

#### 2.2.1. Laparoscopic Kasai Portoenterostomy

The patient is placed in the supine position. We then make a Benz incision in the umbilicus to enlarge the orifice as wide as possible, as previously described by our group [7]. Briefly, three 13-mm lines are drawn in the umbilical region in an inverted Y-shape, and three triangular skin flaps are created by cutting along these lines. Longitudinal incisions are made in the linea alba and peritoneum, and a multichannel port with a 5 mm camera port is inserted through the incision. Two 5-mm ports are inserted into the right paraumbilical and the left upper abdomen, and one 3-mm port is inserted into the right upper abdomen. All manipulations are done using 3 mm forceps and 5 mm microbipolar forceps. The area between the right porta hepatis, in which the right anterior branch of the hepatic artery and portal vein enter the hepatic parenchyma, and the left porta hepatis, in which the left branch of the portal vein enters the parenchyma, is dissected for anastomosis. The fibrous tissue in the hilar plate is dissected just before baring the liver parenchyma. The fibrous tissue is not completely resected and is held lightly on the hilar plate after dissection, which we consider to be one of the most important aspects to successful Kasai. After creating a Roux-en-Y limb with exteriorization via the umbilical incision, end-to-side portoenterostomy is performed laparoscopically.

#### 2.2.2. Laparoscopic Revision of Kasai Portoenterostomy

**Figure 1:**
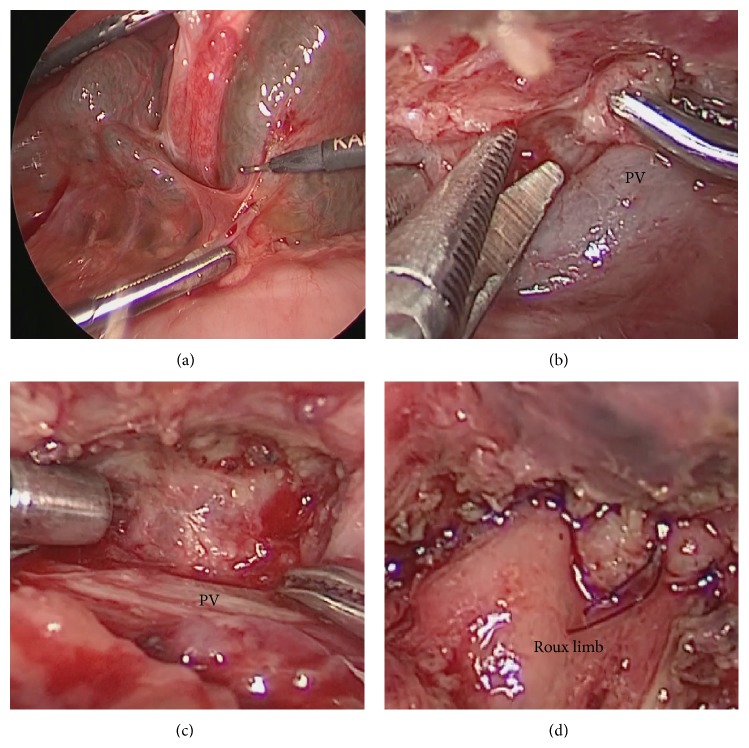
(a) During laparoscopic revision of Kasai portoenterostomy, little adhesion was found around the porta hepatis after the laparoscopic Kasai portoenterostomy. (b) Redissection of the hilar plate during laparoscopic revision of Kasai portoenterostomy. PV: portal vein. (c) The fibrous tissue is transected at the hilar plate before baring the liver parenchyma. The fibrous tissue must not be completely resected and must be held lightly on the hilar plate. (d) End-to-end portoenterostomy is completed laparoscopically after trimming the Roux limb.

In Lap-revision, the ports are placed as in Lap-Kasai without an additional skin incision. During Lap-revision, little adhesion was found around the porta hepatis in all patients after Lap-Kasai (Figure 1(a)). After intraoperative peritoneal adhesiolysis, the anastomosed Roux limb is completely dissected adjacent to the porta hepatis. To avoid unexpected excessive bleeding, the tissues are carefully manupulated using 3-mm forceps and 5-mm microbipolar forceps. The newly developed fibrous extrahepatic tissue is removed (Figure 1(b)) and the hilar plate is superficially dissected just before baring the liver parenchyma (Figure 1(c)). End-to-end portoenterostomy is performed laparoscopically after trimming the tail of the Roux limb (Figure 1(d)).

### 2.3. Postoperative Management

The postoperative management, including antibiotic therapy, steroid therapy, and cholagogue therapy, was similar in all of the patients in this study. Steroid therapy started 1 week after operation. The steroid dose and duration of therapy were slightly different at each patient.

### 2.4. Data Analyses

Data were retrieved from our institutional database and were retrospectively reviewed. The study ended at March 2015, and the status of each patient at that time was recorded. The disappearance of jaundice was defined as a total bilirubin concentration of ≤1.2 mg/dL. Clinical data (patients' characteristics, operation times, blood loss, and surgical outcomes) were compared between open and laparoscopic procedure. This study was approved by our hospital's ethics committee (trial registration number 2014-0406).

### 2.5. Statistical Analyses

Statistical analyses were performed using Fisher's exact test for categorical variables and the Mann–Whitney *U* test for continuous variables. Values of *P* < 0.05 were considered statistically significant.

## 3. Results

### 3.1. The Open and Laparoscopic Kasai Portoenterostomy

The clinical data of Kasai are presented in Table 1. As shown in this table, the open Kasai and laparoscopic Kasai were comparable in terms of the patients' characteristics. The bilirubin concentration was normalized after open and laparoscopic Kasai in 63.1% (41/65) and 66.7% (8/12) of patients, respectively. The median blood loss was significantly less in the Lap-Kasai group than in the open Kasai group (25 mL (range 5–58 mL) versus 50 mL (range 5–363 mL), resp.; *P* = 0.002). However, the median operation time was significantly longer in the Lap-Kasai group than in the open Kasai group (307 min (range 253–448 min) versus 281 min (range 163–395 min), resp.; *P* = 0.036).

### 3.2. The Open and Laparoscopic Revision of Kasai Potoenterostomy

The revision of Kasai was performed in 30.1% (20/65) and 33.3% (4/12) of patients after open and laparoscopic Kasai, respectively.

#### 3.2.1. Characteristics of Patients Who Underwent Laparoscopic Revision of Kasai Portoenterostomy

The characteristics of each patient who underwent Lap-revision are presented in Table 2. Lap-revision was completed in all four patients without conversion or major complications. During Lap-revision, little adhesion was found around the porta hepatis in all patients after Lap-Kasai (Figure 1(a)). The bilirubin concentration was normalized in all four patients following Lap-revision, and three of them were alive with their native liver, without jaundice. One patient, who had an abrupt cessation of bile flow after the bilirubin concentration was normalized (0.9 mg/dL), underwent a living-related liver transplantation.

#### 3.2.2. Comparison between Open and Laparoscopic Revision of Kasai Portoenterostomy

The patients' characteristics and postoperative outcomes are compared between open and laparoscopic revision of Kasai in Table 3. As shown in this table, total bilirubin concentrations before revision were significantly lower in the Lap-revision group than in the open revision group. Operation time, blood loss, and surgical outcomes were similar between open and laparoscopic revision of Kasai.

## 4. Disccussion

The first case of Lap-Kasai was reported by Esteves et al. in 2002 [8]. A recent systematic review and meta-analysis of comparative studies concluded that Lap-Kasai could not replace open Kasai because the 2-year survival rate with the patient's native liver was significantly greater with open Kasai than with Lap-Kasai [9]. However, we think that the excellent visualization of the porta hepatis made possible by laparoscopy enables pediatric surgeons to perform ideal portoenterostomy. Therefore, we have exclusively performed Lap-Kasai since December 2013. Although the number of patients who underwent Lap-Kasai in our study was small, the outcomes were good because 10/12 patients had normal bilirubin concentrations and 11/12 patients had their native liver.

The necessity and indications for revision of Kasai in patients with insufficient biliary drainage after Kasai are controversial [2–6]. According to the Japanese Biliary Atresia Registry (*n* = 2630), revision of Kasai was performed in 21% of the patients and the bilirubin concentration was normalized in 35% of patients who underwent revision of Kasai [2]. To increase the proportion of patients with the disappearance of jaundice, it was suggested that revision of Kasai should be limited to patients with a sudden cessation of bile flow after acheiving sufficient bile drainage following Kasai [2, 3]. In this study, all the patients who underwent Lap-revision had initial good bile drainage. On the other hand, we performed open revision of Kasai, even in patients with initial poor biliary drainage and the native liver was preserved in some of these patients following open revision of Kasai. This is because total bilirubin concentration before revision was significantly higher in the open revision group than in the Lap-revision group. It is difficult to define the most appropriate indications for revision of Kasai. Because laparoscopy offers excellent visualization of the surgical field, accumulated experience of performing Lap-Kasai might enable us to determine what type of porta hepatis benefits most from revision of Kasai. Lap-Kasai might also be useful to define the most appropriate indications for revision of Kasai.

Intraoperative bleeding is a troublesome complication of revision of Kasai. This is because the vessles around the porta hepatis are separated and exposed during Kasai. The amount of blood loss in laparoscopic procedures is generally less than that in open procedures. This advantage of laparoscopic surgery was also observed during Kasai. However, blood loss was similar between laparoscopic and open revision of Kasai in this study. This is because it is difficult to deal with oozing around the porta hepatis, especially in Lap-revision. To promote the use of Lap-revision, it is necessary to prepare for unexpected excessive bleeding in addition to oozing. We used an AirSeal System Insufflator (SurgiQuestInc, Milford, CT, USA) to maintain stable pneumoperitoneum during active bleeding. This system immediately responds to very small changes in intra-abdominal pressure, providing stable pneumoperitoneum and continuous smoke evacuation, even under difficult surgical conditions and constant suction, to ensure good visibility [10]. Future studies should examine whether it is possible to reduce blood loss or operation time using this system.

The longer operation time is the main disadvantage of laparoscopic procedures. Indeed, the operation time was significantly longer for Lap-Kasai than for open Kasai. However, the operation time was similar between laparoscopic and open revision of Kasai, consistent with our surgical findings. In open revision of Kasai, the adhesion around the porta hepatis was dense and adhesiolysis took a long time. By contrast, Lap-revision revealed considerably less adhesion around the porta hepatis after Lap-Kasai. Therefore, it is possible that the disadvantage of repeated laparoscopy in terms of operation time might be canceled by the reduction in adhesion caused by the initial laparoscopic procedure. Some surgeons hesitate to perform revision of Kasai because repeated abdominal procedures are thought to promote adhesion and increase the surgical risks during subsequent LT [5, 6]. Indeed, the time required for hepatectomy at LT was significantly longer in patients who underwent open revision of Kasai than in patients who did not undergo open revision of Kasai in this study (Table 4). If surgeons recognize the feasibility of Lap-revision, it could be regarded as the second standard therapy for BA owing to the reduced severity of adhesion following laparoscopic procedures. If so, many patients with BA might benefit from revision of Kasai and preservation of their native liver. In this study, only one patient underwent LT after Lap-revision. During LT, it was found that the adhesion was less dense than usual, and the time required for hepatectomy during LT was shortest in this patient compared with the other patients who underwent LT in this study (Table 4). However, studies involving a larger number of patients are needed to confirm this advantage of laparoscopic procedure.

In this study, it is a problem that the open and laparoscopic groups were not selected from the patients in the same period. However, we consider that the two groups were comparable because the two groups did not differ in the patients' characteristics and preoperative conditions at the time of Kasai.

In conclusion, Lap-revision  is a feasible and effective procedure for the treatment of BA. By using this technique, the native liver could be preserved in some patients with BA. Moreover, if LT is subsequently required, the adverse effects of Lap-revision might be less than those of open revision of Kasai. Accordingly, we believe that Lap-revision might herald a new era for the treatment of BA. However, studies invovling a larger number of patients and a longer follow-up time are needed to confirm the advantages and disadvantages of this technique.

## Figures and Tables

**Table 1:** The data of open and laparoscopic Kasai portoenterostomy with biliary atresia.

	Open Kasai	Lap-Kasai	*P* value
	*n* = 65	*n* = 12	
Sex (male), *n* (%)	26 (40%)	3 (25%)	0.32
Age at Kasai (days)	66 (32–144)	53 (41–77)	0.002^*∗*^
Weight (g)	4600 (2164–6755)	4273 (3804–5942)	0.36
Tbil concentration before Kasai (mg/dL)	7.5 (3.9–23.6)	6.7 (4.7–11)	0.078
Operation time (min)	281 (163–395)	307 (253–448)	0.036^*∗*^
Blood loss (mL)	50 (5–363)	25 (5–58)	0.002^*∗*^
Disappearance of jaundice after Kasai, *n* (%)	41 (63.1%)	8 (66.7%)	0.43
Revision of Kasai portoenterostomy, *n* (%)	20 (30.1%)	4 (33.3%)	0.86
Status at the last followup, *n* (%)			
Survive with native liver	41 (63.1%)	11 (91.7%)	
Survive with LT	23 (35.4%)	1 (8.3%)	
Died	1 (1.5%)	0	

Values are presented as the *n* (%) or median (range).

^*∗*^ *P* < 0.05.

Kasai: Kasai portoenterostomy; Lap: laparoscopic; Tbil: total bilirubin; LT: liver transplantation.

**Table 2:** Characteristics of patients who underwent laparoscopic revision of Kasai portoenterostomy for the treatment of biliary atresia.

Case	Age at Kasai (days)	Interval between Kasai and revision (days)	Lowest Tbil concentration after Kasai (mg/dL)	Tbil concentration before revision (mg/dL)	Indication for revision	Operation time at revision (min)	Blood loss at revision (mL)	Complication of revision	Lowest Tbil concentration after revision (mg/dL)	Status at last follow-up	Follow-up time (mo)
1	51	69	1.3	5.5	Bile flow cessation	276	192	Incisional hernia	0.9	Survive with LT	10
2	41	73	1.8	3.9	Bile flow cessation	261	80	None	0.3	Survive with native liver	9
3	53	97	0.9	2.4	Bile flow cessation	281	221	None	0.5	Survive with native liver	7
4	53	283	0.8	1.1	Recurrent cholangitis	265	25	None	0.6	Survive with native liver	5

Kasai: Kasai portoenterostomy; revision: revision of Kasai portoenterostomy; Tbil: total bilirubin; LT: liver transplantation.

**Table 3:** The data of open and laparoscopic revision of Kasai portoenterostomy with biliary atresia.

	Open revision	Lap-revision	*P* value
	*n* = 20	*n* = 4	
Sex (male), *n* (%)	7 (35%)	1 (25%)	0.7
Age at revision (days)	103 (63–410)	135 (114–336)	0.22
Weight (g)	5360 (3024–7800)	5457 (5238–8997)	0.44
Tbil concentration before revision (mg/dL)	7.5 (3.5–13.7)	3.2 (1.1–5.5)	0.006^*∗*^
Indication of revision, *n* (%)			0.43
Bile flow cessation	18 (90%)	3 (75%)	
Recurrent cholangitis	2 (10%)	1 (25%)	
Operation time (min)	235 (170–400)	271 (261–281)	0.35
Blood loss (mL)	70 (15–1071)	136 (25–221)	0.62
Disappearance of jaundice after revision, *n* (%)	10 (50%)	4 (100%)	0.11
Status at the last follow-up, *n* (%)			
Survived with native liver	7 (35%)	3 (75%)	
Survived with LT	12 (60%)	1 (25%)	
Died	1 (5%)	0	

Values are presented as the *n* (%) or median (range).

^*∗*^ *P* < 0.05.

Revision: revision of Kasai portoenterostomy; Lap: laparoscopic; Tbil: total bilirubin; LT: liver transplantation.

**Table 4:** Time required for hepatectomy during liver transplantation in patients who underwent Kasai portoenterostomy.

Kasai procedure	Number of patients with LT	Time required for hepatectomy during LT (min)	*P* value
Open Kasai	Without open revision, *n* = 11	214 (141–290)	0.036^*∗*^
	With open revision, *n* = 12	269 (144–370)	

Lap-Kasai	With Lap-revision, *n* = 1	121	

Values are presented as the median (range).

^*∗*^ *P* < 0.05.

Kasai: Kasai portoenterostomy; revision: revision of Kasai portoenterostomy; Lap: laparoscopic; LT: liver transplantation.
